# Analysis of the Variability in the Non-Coding Regions of Influenza A Viruses

**DOI:** 10.3390/vetsci5030076

**Published:** 2018-08-25

**Authors:** Jessica Benkaroun, Gregory J. Robertson, Hugh Whitney, Andrew S. Lang

**Affiliations:** 1 Department of Biology, Memorial University of Newfoundland, 232 Elizabeth Ave., St. John’s, NL A1B 3X9, Canada; jb5163@mun.ca (J.B.); hughwhitneynl@gmail.com (H.W.); 2 Wildlife Research Division, Environment and Climate Change Canada, 6 Bruce St., Mount Pearl, NL A1N 4T3, Canada; greg.robertson@canada.ca

**Keywords:** viral evolution, phylogeny, avian influenza

## Abstract

The genomes of influenza A viruses (IAVs) comprise eight negative-sense single-stranded RNA segments. In addition to the protein-coding region, each segment possesses 5′ and 3′ non-coding regions (NCR) that are important for transcription, replication and packaging. The NCRs contain both conserved and segment-specific sequences, and the impacts of variability in the NCRs are not completely understood. Full NCRs have been determined from some viruses, but a detailed analysis of potential variability in these regions among viruses from different host groups and locations has not been performed. To evaluate the degree of conservation in NCRs among different viruses, we sequenced the NCRs of IAVs isolated from different wild bird host groups (ducks, gulls and seabirds). We then extended our study to include NCRs available from the National Center for Biotechnology Information (NCBI) Influenza Virus Database, which allowed us to analyze a wider variety of host species and more HA and NA subtypes. We found that the amount of variability within the NCRs varies among segments, with the greatest variation found in the HA and NA and the least in the M and NS segments. Overall, variability in NCR sequences was correlated with the coding region phylogeny, suggesting vertical coevolution of the (coding sequence) CDS and NCR regions.

## 1. Introduction

Influenza A viruses (IAVs) are most well known for their circulation in humans, causing yearly seasonal epidemics and occasional pandemics, and for outbreaks in poultry. However, the viral strains responsible for these infections represent only a small portion of the total known IAV diversity, most of which is maintained in the natural wild bird reservoir.

IAVs are members of the family Orthomyxoviridae and have segmented negative-sense single-stranded RNA genomes. The viral genome is composed of eight segments that encode at least 11 proteins, depending on the strain. Each segment has non-coding regions (NCRs) at both ends. The 3′ NCR contains 12 conserved nucleotides followed by a segment-specific region of variable length. The 5′ NCR contains 13 conserved nucleotides and a segment-specific region of variable length. The two conserved regions are partially complementary [1,2,3] and form a panhandle-like structure [4,5,6] involved in the transcription and replication steps of the viral life cycle [7,8,9]. Mutagenesis studies identified residues within the NCRs that are required for the viral polymerase complex to bind and initiate transcription [10,11]. However, a binding preference for the 5′ NCR was demonstrated [12]. There is also a stretch of uridines at the 5′ end that is involved in the polyadenylation of viral mRNAs [13,14,15,16]. Further studies have revealed the presence of some variability within the two NCRs at different positions. There is variability in the fourth nucleotide (C or U) in the 3′ end of all RNA segments [1], which affects the promoter and could play a role in differential regulation of transcription and replication [17]. Non-conserved nucleotides located in the segment-specific regions have also been shown to play an important role in viral replication [18]. Mutagenesis studies have revealed the importance of NCRs for the incorporation of the eight different gene segments during viral assembly [19,20,21,22]. These packaging signals can influence the reassortment of genes during co-infections of different strains by preventing their incorporation during the virus genome packaging [20,23].

Amplification of complete IAV genomes with universal primers was initially designed based on the conserved nature of NCRs [24], but amplification biases have been reported [25,26]. To date, a thorough analysis of IAV NCRs from different hosts and across segments has not been performed. We hypothesized that the NCRs within both the conserved and the segment-specific regions may vary according to the original host species. To test our hypothesis, we characterized the true NCRs of IAV genomes from different wild bird species by the rapid amplification of cDNA ends (RACE) method and then expanded our analyses to include additional viral subtypes, hosts and geographic origins.

## 2. Materials and Methods

### 2.1. NCR Sequence Determination

NCR sequences from previously identified and sequenced viruses originating from three different wild bird host groups (ducks, gulls and seabirds) were determined: A/domestic duck/Newfoundland/MW668/2010(H1N1), A/American black duck/Newfoundland/GR679/2011(H3N2), A/herring gull/Newfoundland/GR578/2011(H13N6), A/herring gull/Newfoundland/GR848/2011(H13N6), A/common murre/Newfoundland/AB318/2011(H1N2), and A/common murre/Newfoundland/AB324/2011(H1N2). From these six viruses, we determined 92 NCR sequences, comprising 46 3′ and 46 5′ NCRs.

Viral genomic RNA was extracted from allantoic fluids of specific pathogen free eggs inoculated with the different viruses using the Trizol reagent (Thermo Fisher Scientific, Waltham, MA, USA). Primers were designed to efficiently amplify the 3′ and 5′ NCRs of the targeted viruses based on the full genomic sequences of viruses that were available from previous studies (Supplementary Table S1).

For the 3′ NCRs, genomic RNAs were polyadenylated with poly(A) polymerase (2 U µL^−1^) (Thermo Fisher Scientific) in the presence of MnCl_2_ (2.5 mM) and ATP (1 mM) in 1X reaction buffer and a final volume of 50 µL. Reactions were incubated at 37 °C for 30 min and the resulting RNA used as the input for the 3′ RACE System for Rapid Amplification of cDNA Ends (Thermo Fisher Scientific). For the 5′ NCRs, the 5′ RACE System for Rapid Amplification of cDNA Ends (Thermo Fisher Scientific) was used according to the manufacturer’s recommendations. The resulting amplicons were purified and sequenced using Sanger chemistry at The Center for Applied Genomics (Toronto, ON, Canada).

### 2.2. Sequence Analyses

The 92 NCRs determined from our samples were compared with NCRs retrieved from the influenza resource database (http://www.ncbi.nlm.nih.gov/genomes/FLU/FLU.html), chosen to represent different viral subtypes, host species, geographic origins, and collection dates. Where possible, all available or a high number of nucleotide sequences of complete gene segments were downloaded from the database. The number of NCRs analyzed are indicated in Figure 3. Sequence alignments were performed using MUSCLE, implemented in MEGA version 6 [27]. Multiple alignments were then edited in Geneious version 8 [28]. The CDS regions of segments that had complete NCRs were removed from the sequence, with only the NCRs kept for further analysis. Complete segments without NCRs were removed from the analysis.

Pairwise genetic distance matrices determined with the Maximum Composite Likelihood method, generated by MEGA, were used for classical multi-dimensional analysis (MDS) in R [29]. The viral segments were assigned geographic origins based on nucleotide BLAST searches [30] and neighbor-joining phylogenetic analyses in MEGA. A small number of sequences was added in each multiple alignment figures due to space limitation, but the exact number of NCRs originally aligned with MUSCLE are indicated in Figure 3.

## 3. Results

### 3.1. Characterization of NCRs from Different Wild Bird Viruses

**Figure 1:**
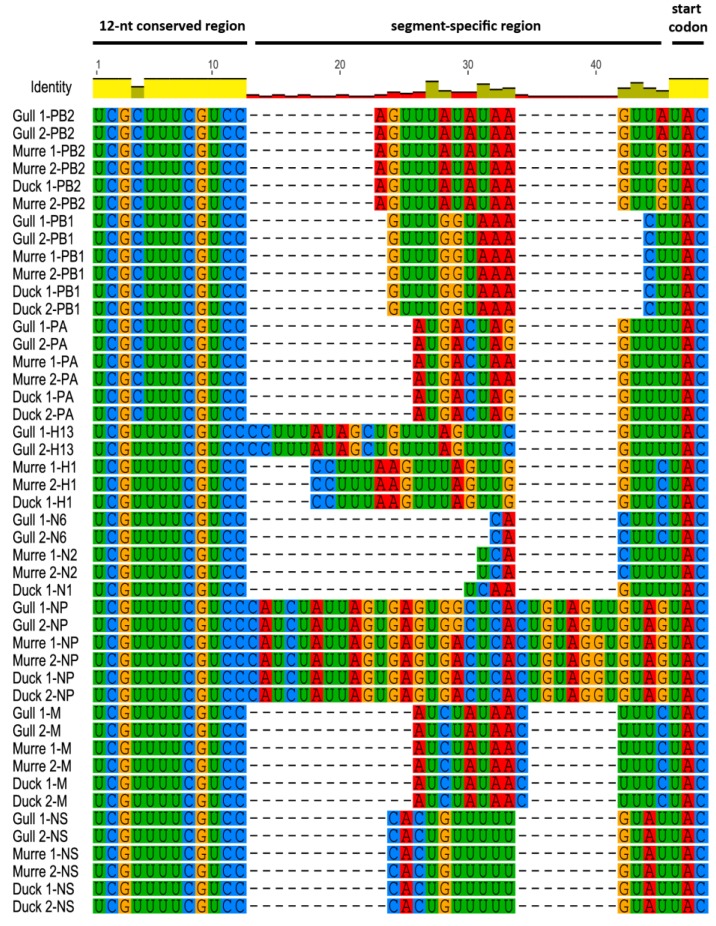
3′ NCRs of wild bird virus segments determined in this study. The multiple alignment of 3′ NCRs was generated with MUSCLE, implemented in Geneious version 8. The RNA sequences are displayed in the 3′ to 5′ orientation corresponding to the packaged RNAs.

**Figure 2:**
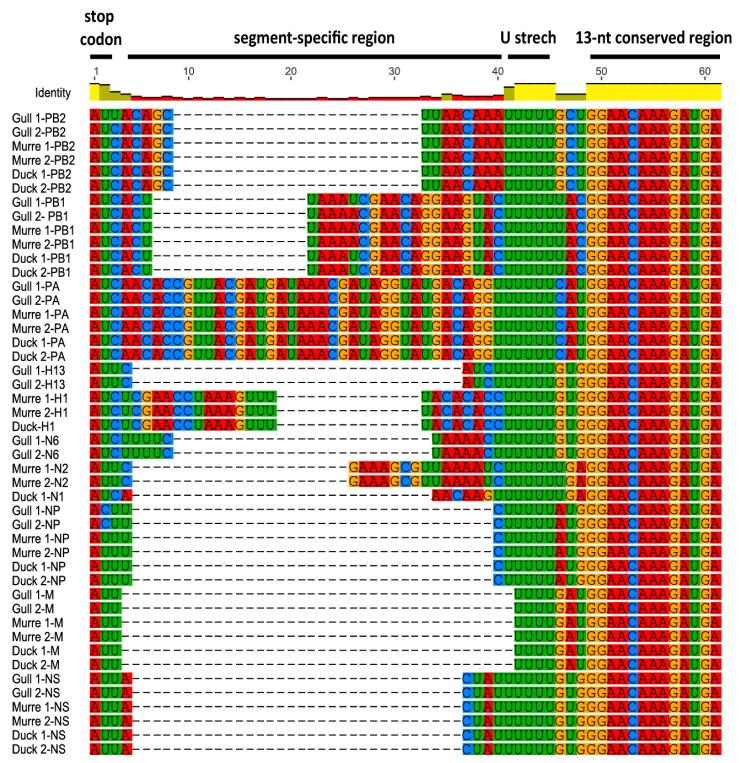
5′ NCRs of wild bird virus segments determined in this study. The multiple alignment of 5′ NCRs was generated with MUSCLE implemented in Geneious version 8. The RNA sequences are displayed in the 3′ to 5′ orientation corresponding to the packaged RNAs.

Comparison of the 3′ and 5′ NCRs we determined by the RACE method showed that the 12- and 13-nt conserved regions, respectively, contained no novel substitutions in any of the different wild bird viruses (Figure 1 and Figure 2), which agrees with previous studies [1,2,3]. Comparison of the 3′ NCRs (Figure 1) showed that the 4th position in the 12-nucleotide conserved region displays the expected variability, with the polymerase gene segments, PB2, PB1 and PA, containing a cytosine residue while the other segments contain a uridine residue, as previously observed [1,17].

For the 3′ segment-specific regions (Figure 1), some of the segments (PB1, M and NS) had identical sequences across all six viruses, but the others showed some differences. The murre and gull PB2 and NP segments were identical whereas the gull viruses differed from the others for these segments. The PA segment was identical in the gull and duck viruses but differed from those in the murre viruses. The HA sequences were identical within subtypes, even for the H1 viruses that came from murres and a duck. Similarly, the NA sequences were identical within the same subtype.

Comparison of the 5′ NCRs showed the conservation of a stretch of five or six uridine residues upstream of the 13-nt conserved region among the different wild bird viruses (Figure 2). Similar to the 3′ NCRs, the segment-specific regions varied among segments, with some of the individual segments’ NCRs conserved among the six viruses and others varying among the viruses (Figure 2). All PB2, PA, M, and NS segments’ NCRs were identical among the viruses. There was a single variation in the PB1 segment, with the murre and one gull virus differing from the other gull virus and the duck viruses. As observed for the 3′ NCRs, the HA and NA segments were the same within subtypes, and the H1 and H2 segments were also identical. 

### 3.2. The Variability in Segment-Specific NCR Sequences Differs among Segments

**Figure 3:**
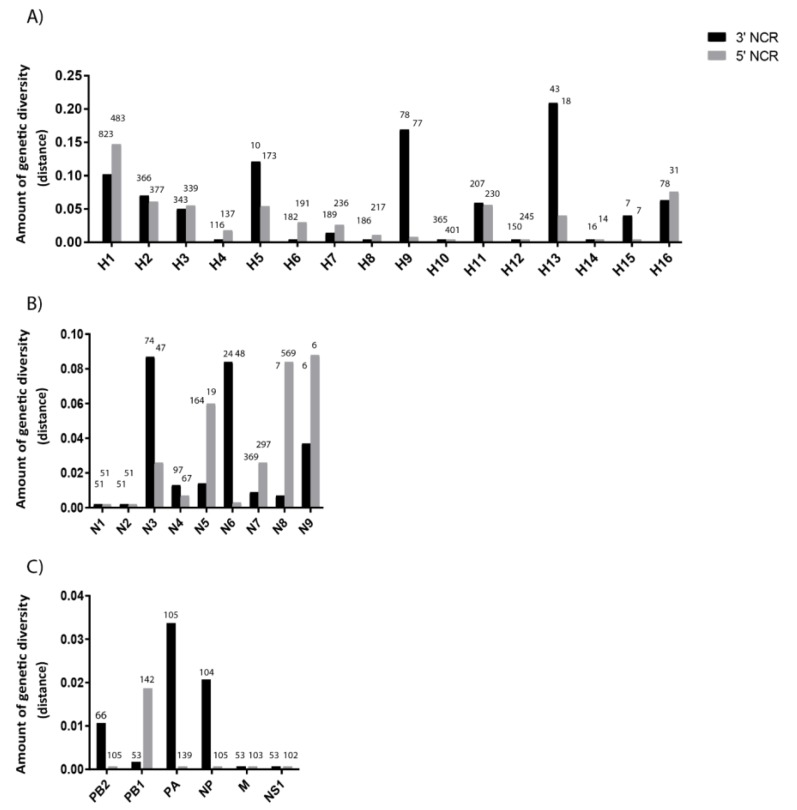
Genetic diversity of 3′ and 5′ NCRs for different segments. (**A**) HA segments of the different subtypes. (**B**) NA segments of the different subtypes. (**C**) “Internal” protein-coding segments. The genetic diversity for each of the NCR regions for the different segments was calculated as mean genetic distance for the NCRs included in our analyses. The number above each bar represents the number of sequences used to calculate the amount of genetic diversity for each segment.

To further investigate the differences observed in the NCRs of our limited set of six viruses, we expanded our analysis of NCR genetic diversity to include viral sequences from a variety of host species and geographic origins retrieved from the National Center for Biotechnology Information (NCBI) Influenza Virus Database (Supplementary Tables S2 and S3). There is no apparent relationship between the number of sequences analyzed and the NCR sequence diversity (Figure 3). The greatest amount of variability in NCRs was observed in the HA segments (Figure 3A), but there is variability among the segments and between 3′ and 5′ NCRs for some subtypes. The H4, H6, H7, H8, H10, H12, and H14 NCRs showed very little variability overall. The H5, H9 and H13 subtypes show greater variability within their 3 ’NCRs relative to their 5′ NCRs, whereas the H1, H2, H3, H11 and H16 were equally variable at the two ends.

The genetic diversity of different NA subtypes’ NCRs (Figure 3B), which originated from a large number of host species and geographic locations, showed that the N3 and N6 subtypes have more diversity at their 3′ ends while the N5, N8, and N9 are more diverse at the 5′ ends. The N1 and N2 viruses showed very little diversity, despite the large assortment of hosts from which viruses were included.

Matching the general pattern observed for IAV segment coding regions, the NCRs of the remaining segments were overall less variable than found for HA and NA (Figure 3C). The M and NS segments showed the lowest diversity, in agreement with the slower evolution rates for these segments’ CDS (e.g., References [31,32]). These differences have been observed across many viruses which is not surprising given that a previous analysis of NCRs, from a limited dataset of human H3N2 viruses circulating worldwide since 1968, found that NCR variability differed among the segments [33].

### 3.3. Patterns of Variability within NCRs Can Be Explained by Viral Host Species and Geographic Origins

To better understand the origin of the segment-specific NCR diversity, we assessed whether the NCRs of viruses from different hosts and geographic origins showed similar patterns of relationships of the respective coding regions (CDS). We used a multi-dimensional scaling (MDS) analysis based on pairwise genetic distance matrices for the NCR sequences, which allowed us to visualize the relationships among NCRs relative to the viral hosts and geographic origins. The results of these analyses were then compared to MDS analyses performed on the respective coding regions. We focused this analysis on segments that showed greater NCR diversity, as described above.

**Figure 4:**
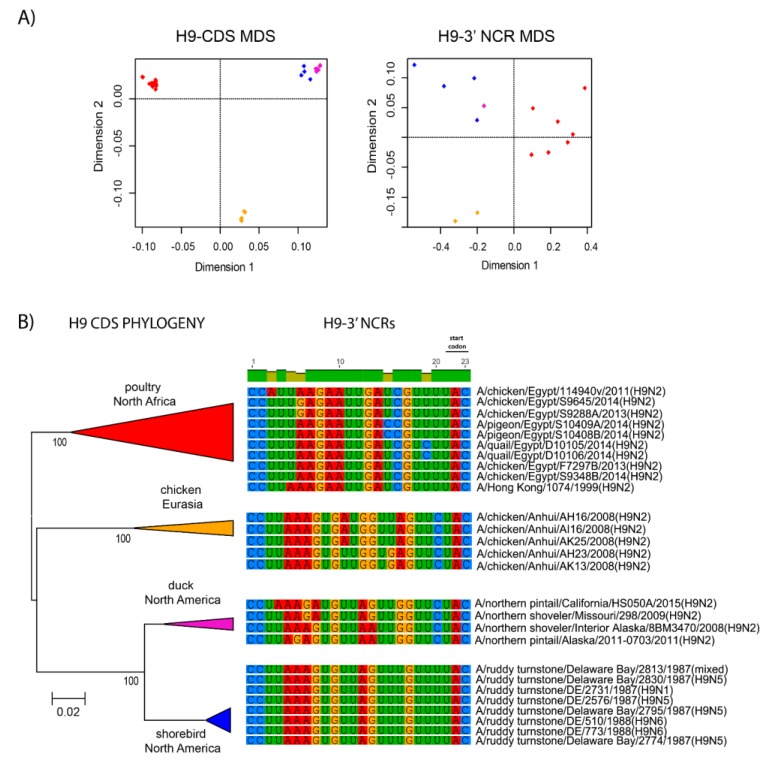
Relationships among H9 3′ NCR and CDS regions for viruses from different host species and geographic locations. The multi-dimensional analysis (MDS) scatter plots (**A**) are based on the pairwise genetic distance matrices from the multiple alignments of complete CDS regions and 3′NCRs. Each dot represents a virus, colored according to their origins as indicated on the CDS-based phylogenetic tree (**B**). Alignments of the 3′ NCRs, shown corresponding to the genomic RNAs, from the viruses used in the analyses are next to the CDS-based neighbor-joining tree for these viruses.

**Figure 5:**
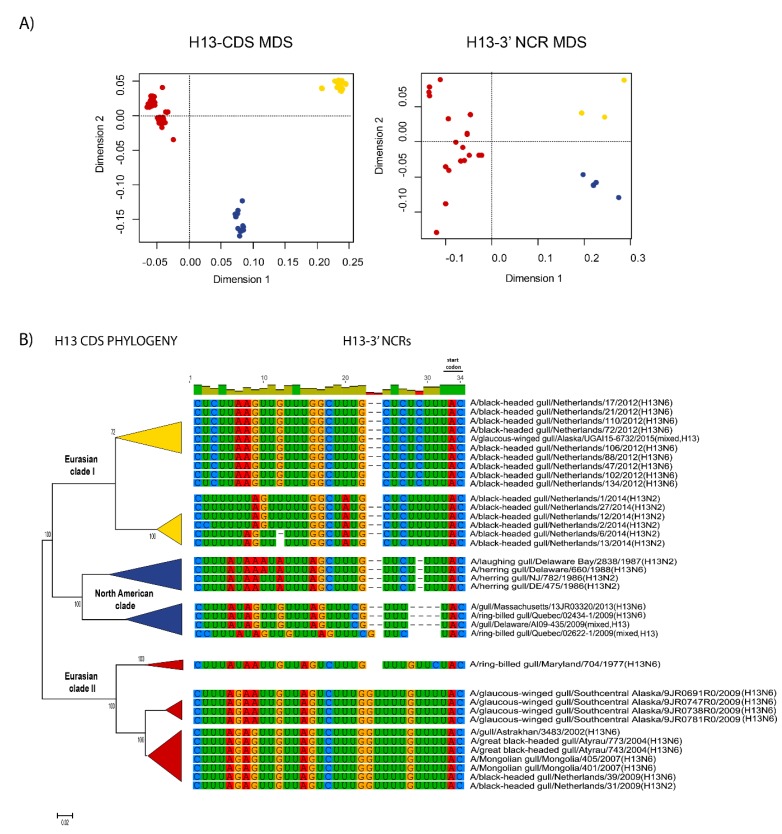
Relationships among H13 3′ NCR and CDS regions for viruses from different host species and geographic locations. The MDS scatter plots (**A**) are based on the pairwise genetic distance matrices from the multiple alignments of complete CDS regions and 3′ NCRs. Each dot represents a virus, colored according to their origins as indicated on the CDS-based phylogenetic tree (**B**). Alignments of the 3′ NCRs, shown corresponding to the genomic RNAs, from the viruses used in the analyses are next to the CDS-based neighbor-joining tree for these viruses.

**Figure 6:**
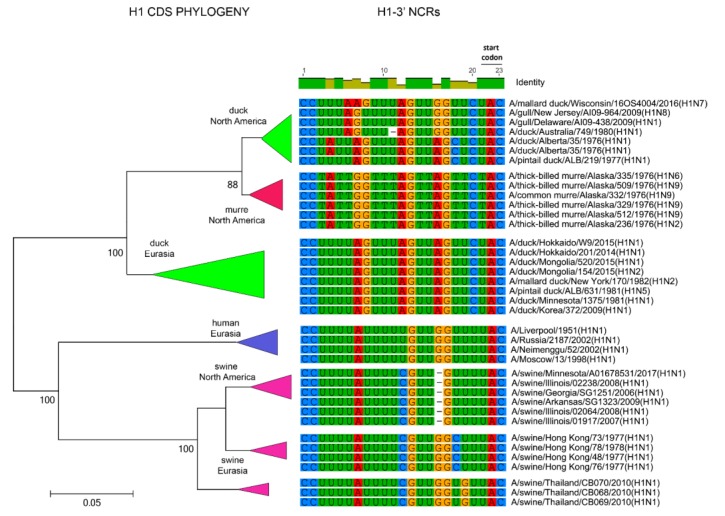
Relationships among H1 3′ NCR and CDS regions for viruses from different host species and geographic locations. Alignments of the 3′ NCRs, shown corresponding to the genomic RNAs, from the viruses used in the analyses are next to the CDS-based neighbor-joining tree for these viruses.

**Figure 7:**
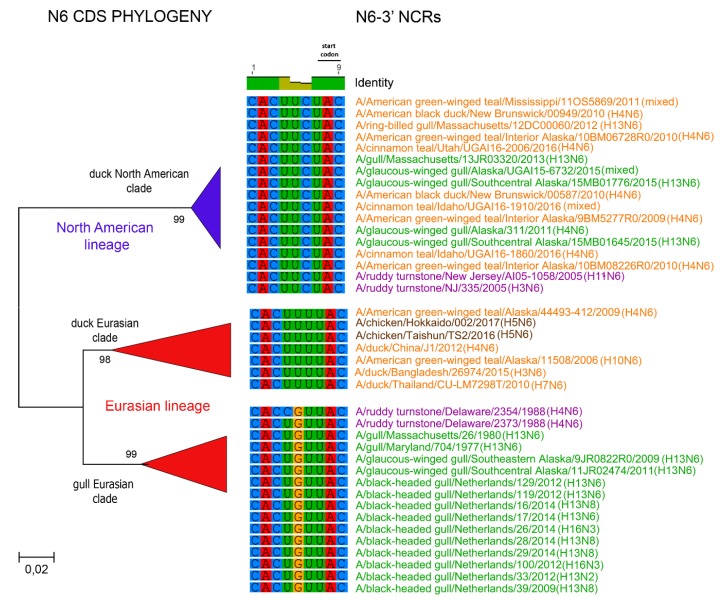
Relationships among N6 3′ NCR and CDS regions for viruses from different host species and geographic locations. Alignments of the 3′ NCRs, shown corresponding to the genomic RNAs, from the viruses used in the analyses are next to the CDS-based neighbor-joining tree for these viruses. To facilitate the distinction of viruses from different hosts, gull viruses are in green, duck viruses are in orange, shorebird viruses are in purple, and chicken viruses are in brown.

**Figure 8:**
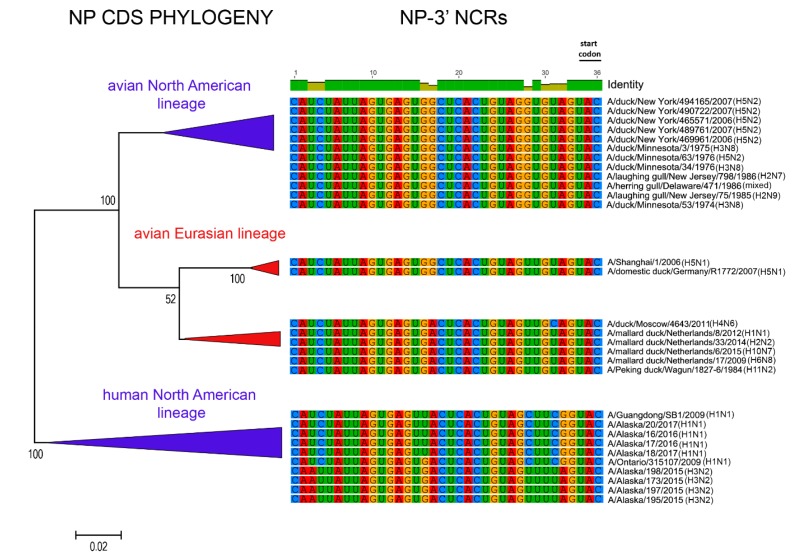
Relationships among NP 3′ NCR and CDS regions for viruses from different host species and geographic locations. Alignments of the 3′ NCRs, shown corresponding to the genomic RNAs, from the viruses used in the analyses are next to the CDS-based neighbor-joining tree for these viruses.

The relationships among the 3′ NCRs and CDS regions of a set of H9 segments were compared (Figure 4). The 3′ NCRs and CDS regions showed similar grouping in the MDS analyses (Figure 4A), corresponding to their CDS phylogenetic clades (Figure 4B). The two North American clade sequences grouped more closely and were more distant from the Eurasian and North African sequences (Figure 4A). This matches the patterns for the CDS phylogeny and 3′ NCR alignments (Figure 4B). The same patterns were observed with the H13 subtype 3′ NCRs in the MDS analyses (Figure 5A), and there was again a clear correspondence between the CDS phylogeny and the NCR sequence relationships (Figure 5B). For the H1 subtype, the CDS phylogeny contained larger host-specific (swine, human and avian) clades that separated into geographic-specific (Eurasian and North American) clades (Figure 6). The correspondence between CDS phylogeny and 3′ NCR similarities was again found for these H1 sequences (Figure 6). A similar pattern was also found for the N6 NA (Figure 7) and NP sequences (Figure 8), where the CDS phylogenies also determined the 3′ NCR relationships. This was true even when the viruses falling within specific clades originated from different hosts (e.g., gulls and ducks) or different continental origins (Eurasia and North America). Overall, these patterns suggest vertical coevolution of the CDS and NCRs.

**Figure 9:**
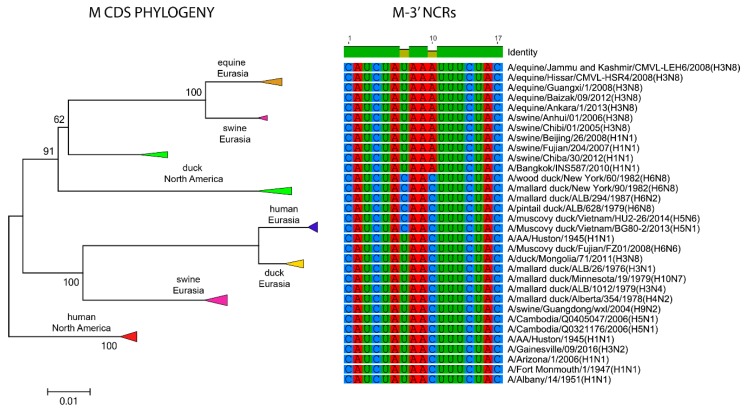
Relationships among M 3′ NCR and CDS regions for viruses from different host species and geographic locations. Alignments of the 3′ NCRs, shown corresponding to the genomic RNAs, from the viruses used in the analyses are next to the CDS-based neighbor-joining tree for these viruses.

Lastly, we looked at the M and NS segments which overall, showed very low NCR diversity (Figure 3C). These two segments share very similar 3′ NCRs amongst different viruses, regardless of the host species or geographic origins (Figure 9, Supplementary Figure S1). Some variations were found for the M segment, for a total of three different M 3′ NCR sequences (Figure 9), but they did not show the same correspondence to the phylogeny of their CDS as observed for the other segments. Comparison of a subset of NS NCRs from various hosts and geographic locations found these all to be identical (Supplementary Figure S1). The conservation of the M and NS NCRs, as mentioned earlier, may be linked to the slower evolutionary rate of the coding regions of these segments.

## 4. Conclusions

We first hypothesized that NCRs may vary according to the host species. Our initial evaluation of the 3′ and 5′ NCRs from viruses isolated from different wild bird hosts showed that the conserved regions are identical among different hosts, but the segment-specific regions showed some variability among the viruses for some segments. This prompted a larger analysis including more sequences, which showed that the amount of variability in the NCRs varies among the different segments and can also vary between the 3′ and 5′ ends, depending on the segment. The overall variability is highest for the HA and NA segments. Examination of 3′ NCRs from HA and NA subtypes with higher variability indicates that the relationships among NCR sequences matches the coding region phylogenies, which generally follow patterns based on the viral host species and geographic origins, and possibly reflects a lack of recombination in these segments. The most conserved NCRs are found on the M and NS segments, which also show the slowest coding sequence evolutionary rates. The exact evolutionary pressures acting on the NCRs and the consequences of sequence changes in these regions, remain to be fully defined.

## Supplementary Materials

The following are available online at http://www.mdpi.com/2306-7381/5/3/76/s1, Figure S1: NS 3′ NCRs from different host species and locations. Hosts of the viruses are indicated on the left, Table S1. List of RACE primers used, Table S2: Number of NCRs analyzed by host, Table S3: Number of NCRs analyzed by geographic location.
