# Supplementary material for: Analysis of the Variability in the Non-Coding Regions of Influenza A Viruses
Source: Vet Sci. 2018 Aug 25;5(3):76. doi: 10.3390/vetsci5030076 (PMC6165000; doi:10.3390/vetsci5030076)
Supplement: Supplementary file 1 [file vetsci-05-00076-s001.zip › vetsci-331630-supplementary.docx]

Supplementary Materials: Analysis of the Variability in the Non-Coding Regions of Influenza A Viruses

Jessica Benkaroun, Gregory J. Robertson, Hugh Whitney and Andrew S. Lang *

**Table S1.** List of Rapid Amplification of cDNA Ends (RACE) primers used.

| **Primer** | **Primer Sequence (5' to 3')** |
| --- | --- |
| **3’ RACE** | |
| 3CM-PB2-1 | CATTAACGGAGCAATCTTGCAG |
| 3CM-PB2-2 | GTGATACCATTACTCTGTCTGATCC |
| 3HG-PB2-1 | CCGGTAGGAATCTGGTCTTGC |
| 3HG-PB2-2 | ACACCATCACCCTATCAGAACC |
| 3DD-PB2-1 | ACTGCTTGTTCCACCAGCC |
| 3DD-PB2-2 | CACGGCCAGAGGTGATACC |
| 3-CM-PB1-1 | CCGGCTGATTCCTGTTTAGTG |
| 3-PB1-2 | ATATCCACTTGGCTCATTGTCC |
| 3-HGDD-PB1-1 | CCGGTTGATTCCTGTTTAATGTC |
| 3-PA-1 | CAATGCAGCCGTTCGGTT |
| 3-CM-PA-2 | ATTATCGATTCACCTCGTTCATC |
| 3-HG-PA-2 | CAGATGCTATTCACCACAGTCC |
| 3-DD-PA-2 | GATTCCACAATTATTGATTCGCC |
| 3-CM-HA-1 | GTGTCCAATAATAGTTCATCCTGCC |
| 3-HG-HA-1 | CGGGTGGAAACTAGTGTATAAGG |
| 3-DD-HA-1 | GTGTCCAGTAATAGTTCATCCTGCC |
| 3-CMDD-HA-2 | TTCAGGCTGCAGAGCTTCC |
| 3-HG-HA-2 | CTCCATTCAAAGAACAGTATGTTCC |
| 3-CM-NA-1 | CATGGCAACTTGAGCTGGAC |
| 3-HG-NA-1 | ATGACATGAGGTGCTGGACC |
| 3-DD-NA-1 | CAATTGTCAACCAGCTAATGCC |
| 3-CM-NA-2 | CGATGTGGAATTCTATCATGTATTG |
| 3-HG-NA-2 | CTCTCCTATTCTAATTGCATTGTCC |
| 3-DD-NA-2 | GCCTGCTAATGTTATTGGAGCC |
| 3-CMDD-NP-1 | GTGGCATCATTCAGATTGGAAT |
| 3-HG-NP-1 | GTGGCGTCATTCAAGTTAGAATG |
| 3-HG-NP-2 | CTGAGCTTGAGTTCGGTGCAC |
| 3-DD-NP-2 | GATGTTCCTCAAGGTATCTGTTCC |
| 3-CM-NP-2 | CCAGATACTTGTTTCTCCTCTCATC |
| 3-M-1 | GCTCACAAGTGGCACATACTAGAC |
| 3-M-2 | CTACGCTGCAGTCCTCGCT |
| 3-NS-1 | AGTATGTCCTGGAAGAGAAGG |
| 3-CMDD-NS-2 | CAGTTAGGTAGCGTGAAGTAGGC |
| 3-HG-NS-2 | CTCTTGACATCTCCTCTGGAGAC |
| **5’ RACE** | |
| 5-CM-PB2-1 | GTAAGGACGCTATTCCAGCAGAT |
| 5-HG-PB2-1 | CAAGGCTGCTAGAGGCCAATAC |
| 5-DD-PB2-1 | CGTGAGGACACTATTCCAGCAG |
| 5-CM-PB2-2 | GGAGTGGAATCTGCGGTATTAAG |
| 5-HG-PB2-2 | GGATGCAGGTTCATTGACAGAAG |
| 5-DD-PB2-2 | GCATTGACTGAAGATCCAGATGA |
| 5-CM-PB2-3 | GATATGGACCAGCATTGAGCAT |
| 5-HG-PB2-3 | GCATCAACGAACTGAGCAATC |
| 5-DD-PB2-3 | GGAGAGAAGGCTAATGTGCTAATTG |
| 5-PB1-1 | CATGAGCATTGGAGTAACAG |
| 5-PB1-2 | GACACACAAATTCAGACAAG |
| 5-PB1-3 | CCACAAAGAGATAGAGTCTGT |
| 5-CM-PA-1 | GTATGTGAGGACCAATGGAACTTC |
| 5-HG-PA-1 | GAGAGCATGATTGAAGCTGAATC |
| 5-DD-PA-1 | GACCGCAATAGGCCAAGTATC |
| 5-CM-PA-2 | GGAGTAGAGGAAGGTTCCATTG |
| 5-HG-PA-2 | GGCTCCATCGGTAAAGTGTG |
| 5-DD-PA-2 | GGAAGGCTCTATTGGGAAGGTAT |
| 5-PA-3 | CTCATTGTTCAGGCACTTAG |
| 5-CMDD-HA-1 | GAATTCAACAACCTAGAGAG |
| 5-HG-HA-1 | GAGTTCAGCCAGGTAGAA |
| 5-CMDD-HA-2 | GAGTTCTATCACAAGTGTGAT |
| 5-HG-HA-2 | GAGCTCCTTCATAAATGTAAT |
| 5-CM-HA-3 | CGGAGTGAAACTAGAGTCGATGG |
| 5-HG-HA-3 | GCTGAATATGCAGAAGAATCG |
| 5-DD-HA-3 | CGGCACATATGACTATCCCAAATAT |
| 5-CM-NA-1 | GGAACGATGATAGCTCTAG |
| 5-HG-NA-1 | GCAAATACTTATGTTCAAAG |
| 5-DD-NA-1 | GTACTAGCTCAAGGAGTGG |
| 5-CM-NA-2 | CGGCTAATTCCAAGTCACAGG |
| 5-HG-NA-2 | GCAGAAACAGACAATCAGTCCG |
| 5-DD-NA-2 | GGATGGACAGAGACGGACG |
| 5-CM-NA-3 | GGTGGACTTCAAATAGTATCGTC |
| 5-HG-NA-3 | GGAATTAATCAGAGGCAGGCC |
| 5-DD-NA-3 | GAGCTAATCAGAGGAAGACCCAAG |
| 5-NP-1 | GTGTCAAGCTTCATCAGA |
| 5-CM-NP-2 | GAGAGAGCAACCATTATGGCA |
| 5-HG-NP-2 | GGAGGAAACACCAACCAAC |
| 5-DD-NP-2 | GGAAACACTGAAGGCAGAACTTC |
| 5-NP-3 | CTGAGATCATAAGGATGATGG |
| 5-M-1 | GTGCCACTTGTGAGCAGATTG |
| 5-M-2 | GGCTAGACAGATGGTGCA |
| 5-M-3 | GCAGCGATTCAAGTGATCCTC |
| 5-CMDD-NS-1 | GCCTTCTCTTCCAGGAC |
| 5-HG-NS-1 | GACATGTCTCCAGAGGAG |
| 5-CMDD-NS-2 | GGACTTGAATGGAATGATAACACAG |
| 5-HG-NS-2 | CTCACCATTACCTTCTCTTCCAGG |
| 5-CMDD-NS-3 | CGCTTGGAGAAGCAGTGATG |
| 5-HG-NS-3 | GGAACAAGAACTGGAGAGAACAAT |

**Table S2.** Number of NCRs analyzed by host.

| **Segment** | **Swine** | **Duck** | **Human** | **Poultry** | **Murre** | **Shorebird** | **Swan/Goose** | **Gull** | **Equine** |
| --- | --- | --- | --- | --- | --- | --- | --- | --- | --- |
| H1 | 191 | 200 | 380 | 11 | 26 | 36 |  |  |  |
| H9 |  | 38 |  | 33 |  |  | 4 | 3 |  |
| H13 |  | 2 |  |  |  |  |  | 41 |  |
| N6 |  |  | 10 |  |  |  |  | 14 |  |
| NP |  | 29 | 16 | 23 |  |  |  | 36 |  |
| M | 16 | 12 | 18 |  |  |  |  |  | 7 |
| NS |  | 16 | 10 | 12 |  |  |  | 15 |  |

**Table S3.** Number of NCRs analyzed by geographic location.

| **Segment** | **Eurasia** | **North America** | **Africa** | **Oceania** | **South America** |
| --- | --- | --- | --- | --- | --- |
| H1 | 239 | 364 | 107 | 78 | 35 |
| H9 | 56 |  | 16 | 6 |  |
| H13 | 19 | 24 |  |  |  |
| N6 | 15 | 9 |  |  |  |
| NP | 56 | 48 |  |  |  |
| M | 23 | 20 |  |  |  |
| NS | 32 | 20 |  |  | 1 |


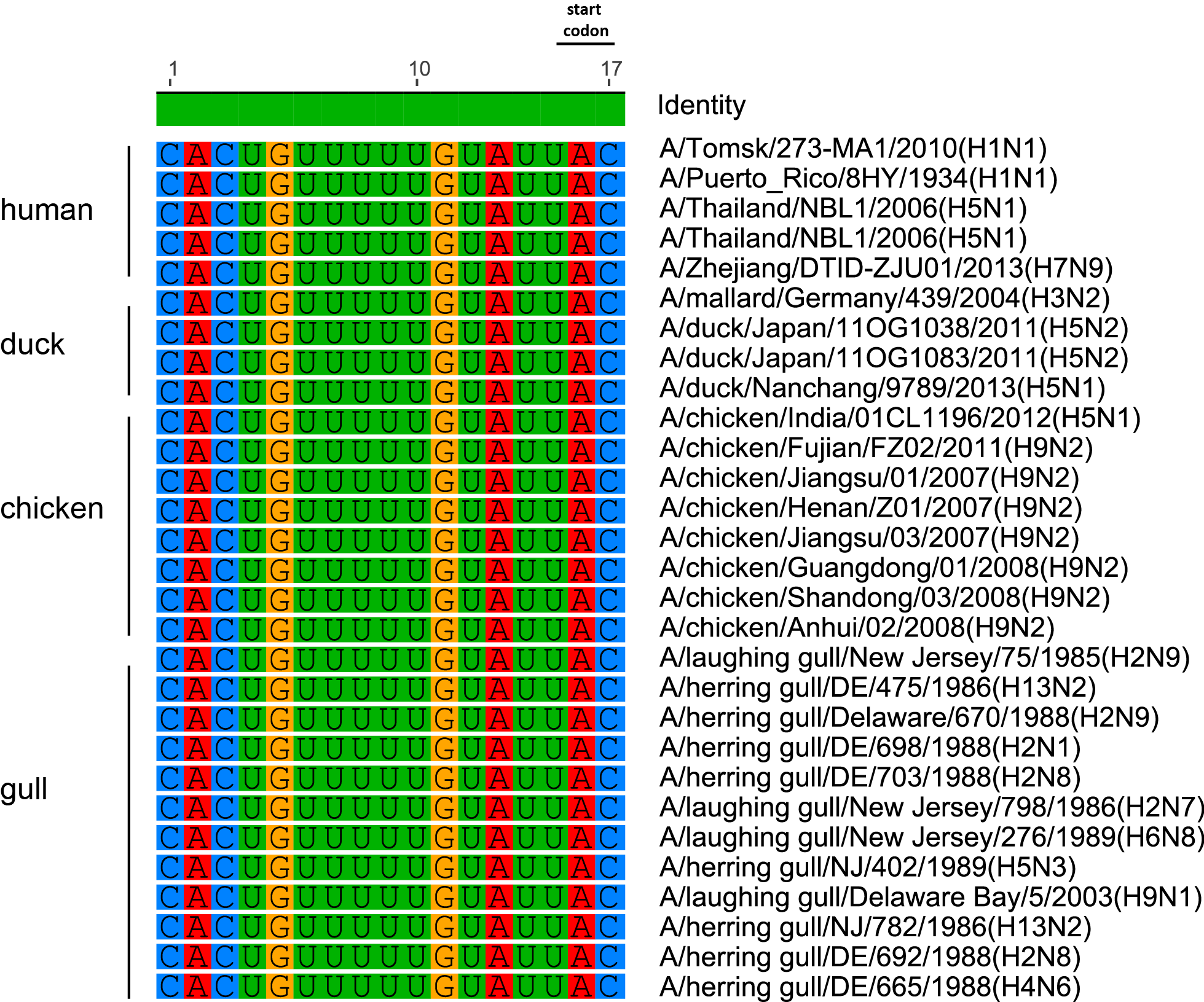


**Figure S1.** NS 3’ NCRs from different host species and locations. Hosts of the viruses are indicated on the left.

© 2018 by the authors. Submitted for possible open access publication under the terms and conditions of the Creative Commons Attribution (CC BY) license (http://creativecommons.org/licenses/by/4.0/).
